# International Medical Congress

**Published:** 1867-09

**Authors:** 


INTERNATIONAL MEDICAL CONGRESS.—The International Congress was opened on the appointed day by M. Bouillaud. The grand amphitheatre was adorned with paintings and flags of all nations, and was entirely filled by members of the Congress. Having called upon the meeting to elect officers, M. Bouillaud was declared elected by acclamation. * * * * * * * *

In the programme of proceedings for Saturday, the 17th of August, we find the announcement of a paper by Prof. Brown-Séquard, under the title of "New Views with regard to the Signs of Cerebral Disease;" and in that for the 27th of August, a paper by Dr. Maxson, of New York, on "Shoulder Presentations;" these are all the contributions from Americans that we see announced.—*Boston Medical and Surgical Journal.*

